# Analysis of Physiological Response during Cardiopulmonary Resuscitation with Personal Protective Equipment: A Randomized Crossover Study

**DOI:** 10.3390/ijerph18137093

**Published:** 2021-07-02

**Authors:** María Fernández-Méndez, Martín Otero-Agra, Felipe Fernández-Méndez, Santiago Martínez-Isasi, Myriam Santos-Folgar, Roberto Barcala-Furelos, Antonio Rodríguez-Núñez

**Affiliations:** 1 CLINURSID Research Group, University of Santiago de Compostela, 15782 Santiago de Compostela, Spain; mariajosefernandezmendez@gmail.com (M.F.-M.); smtzisasi@gmail.com (S.M.-I.); roberto.barcala.furelos@gmail.com (R.B.-F.); Antonio.Rodriguez.Nunez@sergas.es (A.R.-N.); 2 REMOSS Research Group, Faculty of Education and Sports Science, University of Vigo, 36005 Pontevedra, Spain; martinoteroagra@gmail.com (M.O.-A.); m.santos.folgar@gmail.com (M.S.-F.); 3 Pontevedra School of Nursing, University of Vigo, 36004 Pontevedra, Spain; 4 Santiago de Compostela’s Health Research Institute (IDIS), 15706 Santiago de Compostela, Spain; 5 Faculty of Nursing, University of Santiago de Compostela, 15782 Santiago de Compostela, Spain; 6 Department of Obstetrics, Complexo Hospitalario of Pontevedra, Sergas, 36001 Pontevedra, Spain; 7 Pediatric Intensive Care Unit, University Clinical Hospital of Santiago de Compostela, 15706 Santiago de Compostela, Spain

**Keywords:** cardiopulmonary resuscitation, personal protective equipment, physical effort, thermal stress, thermoregulation

## Abstract

The use of personal protective equipment (PPE) is required for the self-protection of healthcare workers during cardiopulmonary resuscitation (CPR) in patients at risk of aerosol transmission of infectious agents. The aim of this study was to analyze the impact of personal protective equipment on physiological parameters during CPR. A randomized, quasi-experimental, crossover design was used. The study was carried out in a training and simulation emergency box and the total sample consisted of 20 healthcare professionals. Two CPR tests were compared with the recommended sequence of 30 chest compressions and 2 ventilations. The duration of each test was 20 min. One of the CPR tests was carried out without using any PPE (CPR_control), i.e., performed with the usual clothing of each rescuer. The other test was carried out using a CPR test with PPE (i.e., CPR_PPE). The main variables of interest were: CPR quality, compressions, ventilations, maximum heart rate, body fluid loss, body temperature, perceived exertion index, comfort, thermal sensation and sweating. The quality of the CPR was similar in both tests. The maximum heart rate was higher in the active intervals (compressions + bag-valve-mask) of the test with PPE. CPR_PPE meant an increase in the perceived effort, temperature at the start of the thermal sensation test, thermal comfort and sweating, as opposed to CPR performed with usual clothing. Performing prolonged resuscitation with PPE did not influence CPR quality, but caused significant physiological demands. Rescuers were more fatigued, sweated more and their thermal comfort was worse. These results suggest that physical preparation should be taken into account when using PPE and protocols for physiological recovery after use should also be established.

## 1. Introduction

The evolution of out-of-hospital medicine towards care and treatment in new environments with viral contamination (EBOLA, Severe Acute Respiratory Syndrome, Middle East Respiratory Syndrome), biological attacks of terrorist origin or environmental/industrial contamination [1] has opened up a new scenario towards the study of safe and efficient resuscitation procedures using personal protective equipment (PPE).

With the emergence of SARS-CoV-2, health care workers have an important risk of aerosol transmission during their procedures [2,3], for this reason the European Resuscitation Council (ERC) [4], the American Heart Association (AHA) [5] included the use of PPE for cardiopulmonary resuscitation (CPR), in any intervention with a suspected or confirmed case of coronavirus disease 2019 (COVID-19) infection.

In the work of emergency medical services (EMS), CPR (compressions and ventilations) is a standard procedure in an emergency room, which leads to a high generation of aerosols and is associated with the risk of infection transmission to medical staff [2]. This explains why the average duration of a work shift for health professionals wearing PPE and not having the possibility of stopping to rest, can be as long as 4 h [6] or more.

CPR is a procedure that causes physical fatigue. Therefore, recommendations for resuscitation indicate relief every two minutes between rescuers [7]. It is also known that resuscitation under special circumstances [8] or in extreme situations (altitude, heat or cold) [9,10,11] can increase anxiety fatigue in untrained people [12] or anyone who has engaged in physically demanding activity beforehand [13]. Scientific evidence also shows that the use of PPE induces changes, both physiologically [14] and in certain technical skills [15,16], such as the lack of perspiration which can accelerate dehydration processes and alter thermoregulation mechanisms. The effect of PPE on resuscitation is not yet consistent. While some studies have found no significant change in the CPR quality [17,18], a recent systematic review suggests that PPE may affect the rate and depth of cardiac compressions (CC) [2]. Despite that, the resuscitation time in all studies reviewed did not exceed 4 min. The reality for emergency departments and pre-hospital emergency departments is that CPR can be prolonged for 30 min or more [19]. Therefore, the objective of this study is to analyze the impact of PPE on physiological demands and CPR quality during prolonged CPR.

## 2. Materials and Methods

### 2.1. Study Design

**Figure 1:**
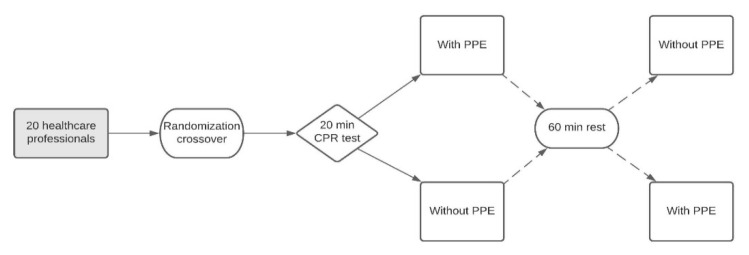
Flow chart of study design.

A randomized, quasi-experimental crossover study was conducted in which two CPR tests were compared with the recommended sequence of 30 chest compressions and 2 ventilations [4,7]. The duration of each test was 20 min (Figure 1).

**Figure 2:**
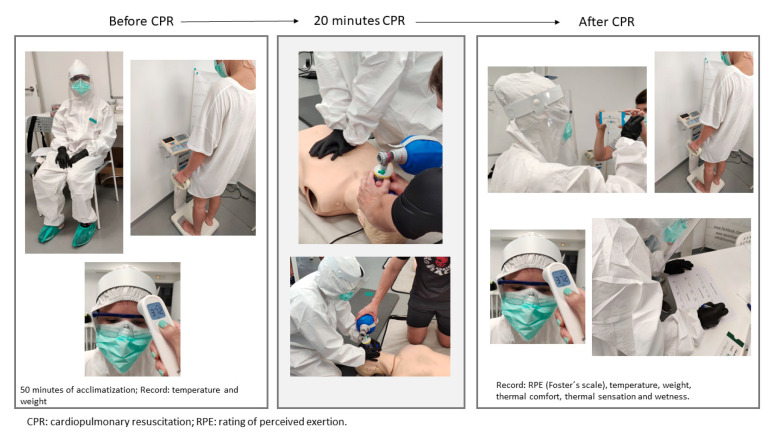
Timeline of the research protocol—before, during and after testing.

- CPR test without PPE (CPR_control): carried out with the usual clothing of each rescuer.
- CPR test with PPE (CPR_PPE): the personal protective equipment consisted of a protective coverall, face shield, goggles, surgical mask, KN95 mask, nitrile gloves and boot swabs (Figure 2).

Although the tests were performed in pairs, only one of the participants was analyzed based on the methodology used in two previous studies of resuscitation in special circumstances [9,10]. The other person was an expert instructor who did not offer feedback or provide additional instructions and his only function was to support the researched participant during the CPR stage. Therefore, for the purposes of this study, only the results of a single rescuer were analyzed.

The resuscitator under study started the test by compressing, so during five cycles of 30 compressions and two ventilations, he performed chest compressions and ventilations with the self-inflating bag (the active phase of CPR), while the supporting instructor attached the mask (the CPR support phase). After these five cycles, there was an exchange of roles, that is, the rescuer passed to the CPR support phase and the instructor carried out the active phase. Thus, the complete 20-min test was divided into intervals of five CPR cycles (these five cycles were approximately 2 min each), in such a way that the sample in this study performed the active phase during the odd intervals (intervals 1, 3, 5, 7, 9 and 11) and the support phase during the even intervals (intervals 2, 4, 6, 8, 10 and 12). Cycles 11 and 12 were only performed by some participants, depending on the mean compression rate (recommended rate: 100–120 compressions/min) [20]. After five cycles, the roles were changed in order to follow the recommendations regarding changing roles every 2 min [7]. The second test was performed after 30 min so as to avoid fatigue bias.

The tests were carried out in an emergency training box, under the following conditions: environmental temperature of 24.8 ± 0.7 °C, and environmental humidity of 66 ± 2%. The environmental temperature was monitored using a TFA Dostmann 30.501 thermometer and hygrometer (Wertheim).

### 2.2. Participants

The participants were recruited from a convenience sample, taking into account the following inclusion criteria: medical personnel trained according to the latest European Resuscitation Council Recommendation Guidelines (ERCGR2015) [20] and the European Resuscitation Council COVID-19 Guidelines [4], who provided 10 effective bag valve mask ventilations during one minute of testing and achieved at least 70% quality of cardiopulmonary resuscitation (QCPR) in one minute of chest compression, who did not present limiting physical injuries to carry out the study and who authorized their participation in writing. The final sample consisted of 20 health professionals (16 nurses, one physiotherapist, one physician and two emergency medical technicians).

This study respected the ethical principles of the Helsinki Convention. Each participant authorized his/her participation and the transfer of the necessary data for this study in writing. The protocol was approved by the ethics committee of the Faculty of Education and Sports Science (University of Vigo), code 10-1020.

### 2.3. Context

#### Retraining in Skills

Before the tests, a refresher session was carried out (CPR review and retraining in a 15-min individual practical session [10,21,22] in accordance with the recommendations of the COVID-19 Guidelines of the European Resuscitation Council [4]. The aim of the session was to standardize skills and become familiar with the manikin and CPR material (i.e., self-inflating bag and face mask). This training was conducted by an instructor accredited by the Spanish CPR Council.

### 2.4. Experimental Procedure and Materials

#### 2.4.1. Physiological Adjustment

The participants accessed the emergency training box where a team of two nurses performed the anthropometric measurements (height, weight, body temperature). After the measurements, they waited 50 min until the beginning of the CPR test [10]. This time was intended for the physiological adjustment to environmental conditions and the PPE. During this timeframe, they were seated in a chair, resting and not making any kind of effort. The aim of the physiological adjustment phase was to simulate a situation in which health professionals who are wearing a PPE at work witness and attend to a cardiac arrest.

#### 2.4.2. Cardiopulmonary Resuscitation (CPR) Test

After the acclimatization period (50 min), a 20-min CPR test was performed on a Laerdal ResusciAnne^®^ manikin (Stavanger, Norway) programmed according to ERCGR2015 [20]. The resuscitation variables were recorded with the SkillReporter QCPR software from Laerdal Medical (Stavanger, Norway). To administer the ventilations, a self-inflating adult-sized bag with a volume of 1500 mL was used. The brand of the bag valve mask was Ambu^®^ Mark IV brand (Ballerup, Denmark) and it had a high efficiency particulate air filter (Clear-Guard™ midi filter with luer port, Intersurgical Ltd., Crane House, Molly Milars Lane, Wokingham, UK). Furthermore, a size 5 clear plastic face shield for adults, Ambu^®^ Mark IV brand (Ballerup, Denmark) was used.

### 2.5. Variables

#### 2.5.1. CPR

The overall CPR quality variable was calculated using the following formula: (overall quality of compressions + percentage of ventilations with adequate volume)/2.

Both the analysis formula for the percentage of overall quality of cardiac compressions and the percentage of effective ventilations are based on previous studies [10,23,24].

1. Chest compression quality

The variables studied in relation to chest compressions were recorded as a percentage and were the following:

- Compressions reaching the target depth: the target depth was considered to be between 5 and 6 cm [20].
- Compressions with a proper re-expansion: those in which the manikin’s chest returned to the starting position before performing the next compression [20].
- Compressions with a proper rate: the recommended rate was followed, that is, between 100 and 120 compressions per minute [20], recorded as a percentage.
- Compressions with a proper hand position: those in which the hands were placed on the lower half of the thorax [20].

The following formula was used to evaluate the compression quality: quality of compressions (%) = (percentage of compressions + percentage of compressions + percentage of compressions)/3.

In addition, the mean velocity of the compressions and the total number of compressions at the end of each test were recorded.

2. Ventilation quality

The active phase of CPR: i.e., with a bag and a mask. During this phase, the variables related to the ventilations were recorded as a percentage:

- Ventilations with adequate volume: those with a volume between 500 and 600 mL [20].
- Insufficient volume ventilation: those with a volume less than 500 mL.
- Excessive volume ventilation: if the record was greater than 600 mL.

CPR support phase: attaching the mask.

Ventilations with effective air inlet: those in which the air inlet and electronic recording of the measurement software are achieved. The percentage was calculated based on the number of effective ventilations (NEV) in relation to the number of total ventilation attempts. The numerical expression is effective ventilations = (NEV × 100)/number of total ventilations.

#### 2.5.2. Physiological Variables

1. Maximum percentage of heart rate during CRP (%HRmax).

Heart rate was measured with the Polar HR Bluetooth sensor H7, (Kempele, Finland), and it was monitored in real time [9,10].

The formula of Karvonen, Kentala, and Mustala [25] was used to calculate the maximum HR as follows: in men: HRmax = 220 − age; and in women: HRmax = 226 − age.

2. Loss of body fluid (LBF).

To calculate the LBF, the body weight in kilograms was recorded before and after each test, using the Tanita BC-418MA high precision scale (Tanita Corporation, Tokyo, Japan) [10].

3. Body temperature.

Body temperature was measured at the frontal level and 1 cm above the left eyebrow [26], at three different times during the PPE test (pre-acclimatization, pre-test and post-test) and at 2 times during the control test (pre-test and post-test). Temperatures were recorded in degrees Celsius and were based on the average of three continuous measurements [10]. All temperature measurements were taken using the Yuwell YT-1 infrared thermometer (Jiangsu Yuyue Medical Equipment and Supply CO., Ltd., Nanjing, China).

4. Ratings of perceived exertion (RPE).

Perceived exertion is the best indicator of the degree of physical exertion since it integrates various information from the muscles, the respiratory and cardiovascular systems and the central nervous system [27]. At the end of each test, the modified Foster scale [28] was used, with a range from 0 (no effort) to 10 (maximum effort).

5. Thermal comfort, thermal sensation and sweating.

For the measurement of thermal comfort, these sensations were subjectively estimated using 20 cm visual analog scales in which the value 0 corresponded to “very cold” and the value 20 to “very hot” [29]. For the measurement of the thermal sensation, the value 0 was registered as “very uncomfortable” and the value 20 as “very comfortable” [29].

The sweating sensation was measured with the Filingeri et al. scale in which the minimum value corresponds to “very dry” and the maximum value is “very wet” [30].

These variables were measured three times during the PPE test (pre-acclimatization, pre-test, and post-test) and two times during the control test (pre-test and post-test).

### 2.6. Statistical Analysis

To determine the sample size, a preliminary estimation analysis was performed assuming the following characteristics: given α = 0.05/Statistical power = 0.95/Effect size = 0.8. The sample size was calculated with the G*Power 3.1.9.2 software (Heinrich-Heine-Universität, Düsseldorf, Germany) using Wilcoxon signed-rank test with matched pairs (a priori compute required sample size). The total sample size obtained was 20 participants.

For the analysis of the variables, the statistical package IBM SPSS Statistics version 20 for Windows (Armonk, NY, USA) was used. The description of continuous variables was carried out with measures of central tendency (mean), dispersion (standard deviation) and confidence estimators (95% confidence intervals). The description of the categorical variables was made through absolute and relative frequencies. Subsequently, the normality of the distributions was analyzed with the Shapiro–Wilk test. For comparisons between the control test and the PPE test, the Student’s *t*-test was used for related samples in the case of the parametric variables, while in the non-parametric variables, the Wilcoxon rank sum test was used. To compare the differences between the tests at different times, the repeated measures analysis of variance (ANOVA) test with Bonferroni correction was used in the case of parametric variables. In the case of non-parametric variables, the Friedman test of repeated measures with Bonferroni correction was used.

## 3. Results

The final sample of this research consisted of 20 health professionals (10 men, 10 women) with a mean age of 31 ± 9 years, height of 164 ± 17 cm and weight of 71 ± 16 kg. Eight had an optimal physical condition, eight had a normal physical condition and four had a bad physical condition.

The comparative results of CPR quality variables are shown in Table 1. No significant differences were observed when comparing the PPE test and control test in any of the variables analyzed (*p* > 0.05).

**Figure 3:**
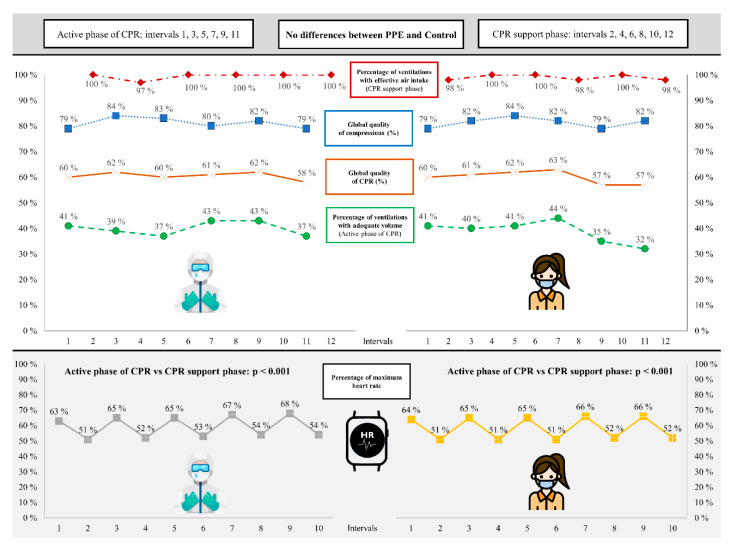
Comparison of compression, ventilation, overall quality of cardiopulmonary resuscitation (CPR) and percentage of maximum heart rate segregated by intervals according to the use of personal protective equipment (PPE).

The results of CPR segregated by intervals are shown in Figure 3. No differences were observed when comparing the intervals with each other in each of the tests or when comparing the PPE test with the control test (*p* < 0.05).

The results of HRmax segregated by intervals are also shown in Figure 3. No significant differences were observed when comparing the PPE test with the control test in each interval (*p* > 0.05). However, when analyzing the intervals in each test, significant differences were observed when we compared the active intervals with the support intervals (*p* < 0.001). In this sense, HRmax in the active intervals ranged between 63–68% in the case of the PPE test and 64–66% in the control test. In contrast, in the support intervals, HRmax in the PPE test was between 51–54% and between 51–52% in the control test.

**Figure 4:**
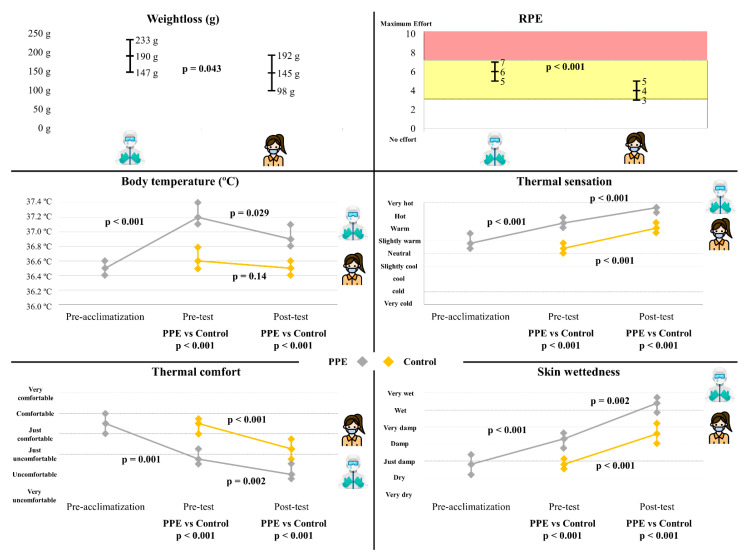
Comparison of weightloss, ratings of perceived exertion (RPE), body temperature and perception variables by time and use of personal protective equipment (PPE).

The physiological results and the perception variables are shown in Figure 4. The PPE test presents a significantly greater weight loss (190 ± 91 g) than the control test (145 ± 100 g) with a value of *p* = 0.043.

Perceived fatigue was also greater when performing the PPE test (6 ± 2) with respect to the CPR_control (4 ± 2) (*p* < 0.001) (Figure 4).

There is a rise in temperature during the acclimatization of the PPE Test (from 36.5 ± 0.3 °C to 37.2 ± 0.4 °C; *p* < 0.001), then there is a decrease to 36.9 ± 0.3 °C at the end of the test (*p* = 0.029). In the control test, by contrast, the temperature remains stable at the end of the test (from 36.6 ± 0.3 °C to 36.5 ± 0.2 °C; *p* = 0.14). The body temperature with PPE was significantly higher before starting and at the end of the test (*p* < 0.001). In this way, the perceived thermal sensation was greater when using the PPE (19 ± 1: “between the sensation of hot and very hot”; *p* < 0.001) showing an upward trend in both tests (*p* < 0.001). In the same way, perceived thermal comfort was lower when using PPE (4 ± 4: “uncomfortable”; *p* < 0.001) showing a downward trend in both tests (*p* ≤ 0.002). Finally, the perceived sweating was higher when using PPE (18 ± 3: “between wet and very wet”; *p* < 0.001) showing an upward trend in both tests (*p* ≤ 0.002) (Figure 4).

## 4. Discussion

The objective of this study was to analyze the impact of personal protective equipment on the physiological parameters of the rescuer and on the quality of the CPR during resuscitation. The main findings of this study were: (a) CPR performance was not affected by the fact that rescuers wore PPE, (b) fatigue, temperature, thermal sensation and sweating increases when performing CPR with PPE, while thermal comfort decreases.

Regarding the quality of CPR, a systematic review reveals that the use of PPE reduces the quality of compressions when compared to CPR in which the participants do not wear PPE [2]. However, these studies have been performed in single-rescuer situations, without the possibility of rest periods and in resuscitations of short duration (2–4 min) [16,31,32]. In addition, only one of these studies used the 30:2 protocol [16]. On the other hand, Kienbacher et al. conducted a study with a 30:2 protocol of a 12 min duration performed in pairs changing roles every 2 min. This study did not show a lower quality of CPR when EPP was used, compared to not using EPP [33]. Our study confirms the message of Kienbacher et al. by providing evidence of a longer resuscitation duration. The absence of differences in the quality of CPR when using PPE compared to not using PPE reinforces the message that healthcare professionals should protect themselves with PPE in risky situations, as the quality of care is not adversely affected.

Due to the decrease in CPR quality evidenced in their systematic review, Sahu et al. recommend finding ways to improve CPR without compromising on the safety of the healthcare worker [2]. Both Kienbacher et al. and this study provide evidence for the non-inferiority of resuscitation in longer scenarios [33]. This study shows a significant decrease in HR during the CPR support phase. Perhaps the application of protocols with more than one rescuer in which recovery periods can be performed is the main key to ensuring quality resuscitation and the safety of the healthcare worker.

The increase in HR during the active phase of CPR should be analyzed along with the RPE and the decrease in weight due to the loss of body fluids. There is an association between temperature and an increase in RPE [34], and in turn between an increase in HR and an increase in RPE [35] as well as between dehydration and early heat fatigue [34].

This study is the first to measure body temperature during CPR with PPE. In this study, we obtain similar temperature results when performing CPR with PPE to those obtained in a 37 °C ambient temperature scenario [10]. To control this rise in temperature and with the activation of the thermoregulatory system, we obtained a greater loss of fluid when our rescuers performed CPR with PPE (after 50 min of acclimatization and 20 min of CPR). In a Spanish study they also obtained greater body fluid losses in the extreme temperature scenario and with statistically significant losses, although, in their case, these values are 10 times greater than ours [10]. Taking into account that the performance deficit in aerobic activities occurs with the dehydration of 3% of body water or a loss of 2% in body mass index [36], a longer resuscitation time, a longer period of acclimatization, previous dehydration or a higher temperature could be limiting factors to maintain the performance of cardiopulmonary resuscitation [10]. During exercise in the heat, sweat production usually exceeds water intake, hence there is water deficit in the body [37]. Therefore, it would be advisable for healthcare professionals to remove PPE after performing CPR and drink fluids in a room at ambient temperature to rehydrate and lower their body temperature so that they can continue their work in suitable conditions.

Regarding the perceived fatigue when performing 20 min of CPR, this was higher when the participants carried out resuscitation with PPE, giving a mean score of 6 following the values of Foster et al. [28], perceiving CPR as “hard”, while in the control test it was “somewhat difficult” (mean score of 4). Performance assessment using perceived stress scales is a widely used tool with a high level of validity for quantifying exercise intensity [36] and is commonly used in resuscitation studies with rescuers [9,10,23,31,38,39]. These differences are similar to those obtained in a previous study in which they compared the quality of cardiopulmonary resuscitation in two environments with different temperatures, at 25 °C and 37 °C, respectively [10]. The increase in RPE reflected when performing CPR with PPE is in concordance with other studies, in which perceived fatigue shows higher values when wearing PPE than when not wearing PPE [16,32,33]. It would be advisable for healthcare professionals to rest after performing CPR with PPE until their sense of fatigue decreases.

This increase in fatigue when performing CPR with PPE could be related to an increase in body temperature [34] and thermal comfort [40]. Thermal sensation and thermal comfort can be modified by skin temperature, in such a way that when exercise is performed in both hot and moderate environmental conditions, thermal discomfort is greater in the hot ones [40]. In addition, thermal comfort during exercise can be modulated by skin humidity, as in the cases of exercise in the heat and profuse sweating, and it has been determined that skin temperature does not have special relevance to determine thermal comfort due to cooling by evaporation of sweat. Sweating does not directly reduce thermal comfort, therefore its accumulation on the skin contributes to thermal discomfort [40]. Even so, the participants in this research were “uncomfortable” and “wet or very wet” when they finished the test wearing the PPE. This is because thermal comfort during hot exercise is strongly influenced by the choice of clothing [41]. This also reinforces the idea of removing PPE after resuscitation and recovering until dry and comfortable to return to work safely.

### Limitations

This investigation has been carried out in a controlled simulation situation with a manikin, so it does not necessarily represent the characteristics of a real victim. The rest period between each test was 60 min. A longer rest period could have obtained different results in the second test. Due to the personal protection measures established during this pandemic, the participants in this research had to wear a surgical mask during the control CPR test, which could reduce comfort for the application of resuscitation skills

## 5. Conclusions

When healthcare professionals wear PPE, they are capable of prolonged CPR of a quality comparable to when it is performed in normal clothing, although this involves a considerable physiological stress. Therefore, it is important to be aware of the physiological impact of prolonged exertion. CPR training wearing PPE is necessary for rescuers in order to understand this special circumstance and it might also improve their physical condition. It makes sense to remove PPE, rehydrate and rest in a cool environment to reduce body temperature and feel comfortable.

## Figures and Tables

**Table 1:** Comparison of the compression and ventilation variables and the overall CPR quality during 20 min of CPR according to the clothing used (with or without PPE).

*N* = 20	CPR with PPE		CPR Control		
	Mean (SD)	CI	Mean (SD)	CI	
**Compressions**					
Overall quality of compressions, in percentage terms	81 (14)	75–88	81 (13)	75–87	*p* = 0.82
Total number of compressions	868 (50)	844–891	867 (48)	845–890	*p* = 1.00
Percentage of compressions that reach the correct depth	62 (32)	47–77	61 (32)	46–76	*p* = 0.74
Percentage of compressions with correct reexpansion	89 (19)	81–98	88 (19)	78–97	*p* = 0.52
Percentage of compressions with correct rhythm	75 (31)	61–90	77 (28)	64–90	*p* = 1.00
Percentage of compressions with correct hand position	99 (2)	98–100	100 (1)	99–100	*p* = 0.96
**Ventilations**					
**Active phase of CPR (compressions and ventilations)**					
Total number of ventilations	58 (4)	56–59	57 (4)	55–59	*p* = 0.48
Percentage of ventilations with insufficient volume	38 (39)	20–57	36 (39)	18–55	*p* = 0.62
Percentage of ventilations with correct volume	40 (34)	25–56	40 (36)	23–56	*p* = 0.85
Percentage of ventilations with excessive volume	21 (35)	5–38	24 (39)	6–42	*p* = 0.75
**CPR support phase (attaching the mask)**					
Total number of ventilations	52 (2)	51–53	52 (2)	50–53	*p* = 0.86
Percentage of ventilations with effective air intake	99 (1)	98–100	99 (2)	98–100	*p* = 0.79
**Overall quality of CPR, in percentage terms**	61 (19)	52–70	61 (20)	51–68	*p* = 0.93

CPR: cardiopulmonary resuscitation; PPE: personal protective equipment; SD: standard deviation. CI: confidence intervals.

## Data Availability

Not applicable.

## References

[1] Sprague R.M., Ladd M., Ashurst J.V. EMS Resuscitation During Contamination While Wearing PPE. http://www.ncbi.nlm.nih.gov/books/NBK534092/.

[2] Sahu A.K., Suresh S., Mathew R., Aggarwal P., Nayer J. (2020). Impact of personal protective equipment on the effectiveness of chest compression—A systematic review and meta-analysis. Am. J. Emerg. Med..

[3] Malhotra N., Gupta N., Ish S., Ish P. (2020). COVID-19 in intensive care. Some necessary steps for health care workers. Monaldi Arch. Chest Dis..

[4] Nolan J.P., Monsieurs K.G., Bossaert L., Böttiger B.W., Greif R., Lott C., Madar J., Olasveengen T.M., Roehr C.C., Semeraro F. (2020). European Resuscitation Council COVID-19 guidelines executive summary. Resuscitation.

[5] Edelson D.P., Sasson C., Chan P.S., Atkins D.L., Aziz K., Becker L.B., Berg R.A., Bradley S.M., Brooks S.C., Cheng A. (2020). Interim Guidance for Basic and Advanced Life Support in Adults, Children, and Neonates With Suspected or Confirmed COVID-19: From the Emergency Cardiovascular Care Committee and Get With The Guidelines-Resuscitation Adult and Pediatric Task Forces of the American Heart Association. Circulation.

[6] Tabah A., Ramanan M., Laupland K.B., Buetti N., Cortegiani A., Mellinghoff J., Morris A.C., Camporota L., Zappella N., Elhadi M. (2020). Personal protective equipment and intensive care unit healthcare worker safety in the COVID-19 era (PPE-SAFE): An international survey. J. Crit. Care.

[7] Monsieurs K.G., Nolan J.P., Bossaert L.L., Greif R., Maconochie I.K., Nikolaou N.I., Perkins G.D., Soar J., Truhlář A., Wyllie J. (2015). European Resuscitation Council Guidelines for Resuscitation 2015: Section 1. Executive summary. Resuscitation.

[8] Truhlář A., Deakin C.D., Soar J., Khalifa G.E.A., Alfonzo A., Bierens J.J.L.M., Brattebø G., Brugger H., Dunning J., Hunyadi-Antičević S. (2015). European Resuscitation Council Guidelines for Resuscitation 2015: Section 4. Cardiac arrest in special circumstances. Resuscitation.

[9] Carballo-Fazanes A., Barcala-Furelos R., Eiroa-Bermúdez J., Fernández-Méndez M., Abelairas-Gómez C., Martínez-Isasi S., Murciano M., Fernández-Méndez F., Rodríguez-Núñez A. (2020). Physiological demands of quality cardiopulmonary resuscitation performed at simulated 3250 meters high. A pilot study. Am. J. Emerg. Med..

[10] Barcala-Furelos R., Fernández-Méndez M., Cano-Noguera F., Otero-Agra M., Morán-Navarro R., Martínez-Isasi S. (2020). Measuring the physiological impact of extreme heat on lifeguards during cardiopulmonary resuscitation. Randomized simulation study. Am. J. Emerg. Med..

[11] Martin-Conty J.L., Martin-Rodríguez F., Criado-Álvarez J.J., Romo Barrientos C., Maestre-Miquel C., Viñuela A., Polonio-López B., Durantez-Fernández C., Marcos-Tejedor F., Mohedano-Moriano A. (2020). Do Rescuers’ Physiological Responses and Anxiety Influence Quality Resuscitation under Extreme Temperatures?. Int. J. Environ. Res. Public Health.

[12] Abelairas-Gómez C., Barcala-Furelos R., Szarpak L., García-García Ó., Paz-Domínguez Á., López-García S., Rodríguez-Núñez A. (2017). The effect of strength training on quality of prolonged basic cardiopulmonary resuscitation. Kardiol. Pol..

[13] Barcala-Furelos R., Abelairas-Gomez C., Romo-Perez V., Palacios-Aguilar J. (2013). Effect of physical fatigue on the quality CPR—A water rescue study of lifeguards: Physical fatigue and quality CPR in a water rescue. Am. J. Emerg. Med..

[14] Loibner M., Hagauer S., Schwantzer G., Berghold A., Zatloukal K. (2019). Limiting factors for wearing personal protective equipment (PPE) in a health care environment evaluated in a randomised study. PLoS ONE.

[15] Malysz M., Dabrowski M., Böttiger B.W., Smereka J., Kulak K., Szarpak A., Jaguszewski M., Filipiak K.J., Ladny J.R., Ruetzler K. (2020). Resuscitation of the patient with suspected/confirmed COVID-19 when wearing personal protective equipment: A randomized multicenter crossover simulation trial. Cardiol. J..

[16] Kim T.H., Kim C.H., Shin S.D., Haam S. (2016). Influence of personal protective equipment on the performance of life-saving interventions by emergency medical service personnel. Simulation.

[17] Donoghue A.J., Kou M., Good G.L., Eiger C., Nash M., Henretig F.M., Stacks H., Kochman A., Debski J., Chen J.-Y. (2020). Impact of Personal Protective Equipment on Pediatric Cardiopulmonary Resuscitation Performance: A Controlled Trial. Pediatr. Emerg. Care.

[18] Barcala-Furelos R., Abelairas-Gómez C., Alonso-Calvete A., Cano-Noguera F., Carballo-Fazanes A., Martínez-Isasi S., Rodríguez-Núñez A. (2021). Safe On-Boat Resuscitation by Lifeguards in COVID-19 Era: A Pilot Study Comparing Three Sets of Personal Protective Equipment. Prehosp. Disaster Med..

[19] Song W., Liu Y., Ouyang Y., Chen W., Li M., Xianyu S., Yi S. (2020). Recommendations on cardiopulmonary resuscitation strategy and procedure for novel coronavirus pneumonia. Resuscitation.

[20] Perkins G.D., Handley A.J., Koster R.W., Castrén M., Smyth M.A., Olasveengen T., Monsieurs K.G., Raffay V., Gräsner J.-T., Wenzel V. (2015). European Resuscitation Council Guidelines for Resuscitation 2015: Section 2. Adult basic life support and automated external defibrillation. Resuscitation.

[21] Nishiyama C., Iwami T., Murakami Y., Kitamura T., Okamoto Y., Marukawa S., Sakamoto T., Kawamura T. (2015). Effectiveness of simplified 15-min refresher BLS training program: A randomized controlled trial. Resuscitation.

[22] Adelborg K., Bjørnshave K., Mortensen M.B., Espeseth E., Wolff A., Løfgren B. (2014). A randomised crossover comparison of mouth-to-face-shield ventilation and mouth-to-pocket-mask ventilation by surf lifeguards in a manikin. Anaesthesia.

[23] Barcala-Furelos R., Abelairas-Gomez C., Palacios-Aguilar J., Rey E., Costas-Veiga J., Lopez-Garcia S., Rodriguez-Nunez A. (2017). Can surf-lifeguards perform a quality cardiopulmonary resuscitation sailing on a lifeboat? A quasi-experimental study. Emerg. Med. J. EMJ.

[24] Fungueiriño-Suárez R., Barcala-Furelos R., González-Fermoso M., Martínez-Isasi S., Fernández-Méndez F., González-Salvado V., Navarro-Patón R., Rodríguez-Núñez A. (2018). Coastal Fishermen as Lifesavers While Sailing at High Speed: A Crossover Study. BioMed. Res. Int..

[25] Karvonen M. (1957). The effects of training on heart rate: A longitudinal study. Ann. Med. Exp. Biol. Fenn..

[26] Padilla-Raygoza N., Ruiz-Paloalto M.L., Díaz-Guerrero R., Olvera-Villanueva G., Maldonado A., del Raygoza-Mendoza M.P. (2014). Correlación de mediciones de temperatura corporal con 3 termómetros: Ótico, cutáneo y digital, en niños mexicanos. Enferm. Clín..

[27] Borg G.A. (1982). Psychophysical bases of perceived exertion. Med. Sci. Sports Exerc..

[28] Foster C., Florhaug J.A., Franklin J., Gottschall L., Hrovatin L.A., Parker S., Doleshal P., Dodge C. (2001). A new approach to monitoring exercise training. J. Strength Cond. Res..

[29] Davey S., Reilly M., Newton M., Tipton M. The Reproducibility and Validity of Visual Analogue Scales (VAS) that Assess Thermal Perceptions in Stable and Dynamic, Asymmetric Environments. Proceedings of the 12th International Conference on Environmental Ergonomics.

[30] Filingeri D., Fournet D., Hodder S., Havenith G. (2014). Why wet feels wet? A neurophysiological model of human cutaneous wetness sensitivity. J. Neurophysiol..

[31] Chen J., Lu K.-Z., Yi B., Chen Y. (2016). Chest Compression with Personal Protective Equipment During Cardiopulmonary Resuscitation: A Randomized Crossover Simulation Study. Medicine.

[32] Tian Y., Tu X., Zhou X., Yu J., Luo S., Ma L., Liu C., Zhao Y., Jin X. (2020). Wearing a N95 mask increases rescuer’s fatigue and decreases chest compression quality in simulated cardiopulmonary resuscitation. Am. J. Emerg. Med..

[33] Kienbacher C.L., Grafeneder J., Tscherny K., Krammel M., Fuhrmann V., Niederer M., Neudorfsky S., Herbich K., Schreiber W., Herkner H. (2021). The use of personal protection equipment does not impair the quality of cardiopulmonary resuscitation: A prospective triple-cross over randomised controlled non-inferiority trial. Resuscitation.

[34] González-Alonso J., Coyle E.F. (1998). Efectos fisiológicos de la deshidratación. ¿Por qué los deportistas deben ingerir líquidos durante el ejercicio en el calor?. Apunts. Educ. Física Deport..

[35] Scherr J., Wolfarth B., Christle J.W., Pressler A., Wagenpfeil S., Halle M. (2013). Associations between Borg’s rating of perceived exertion and physiological measures of exercise intensity. Eur. J. Appl. Physiol..

[36] Cheuvront S.N., Kenefick R.W., Montain S.J., Sawka M.N. (2010). Mechanisms of aerobic performance impairment with heat stress and dehydration. J. Appl. Physiol..

[37] Sawka M.N., Montain S.J. (2000). Fluid and electrolyte supplementation for exercise heat stress. Am. J. Clin. Nutr..

[38] Barcala-Furelos R., Szpilman D., Palacios-Aguilar J., Costas-Veiga J., Abelairas-Gomez C., Bores-Cerezal A., López-García S., Rodríguez-Nuñez A. (2016). Assessing the efficacy of rescue equipment in lifeguard resuscitation efforts for drowning. Am. J. Emerg. Med..

[39] Abelairas-Gómez C., Barcala-Furelos R., Mecías-Calvo M., Rey-Eiras E., López-García S., Costas-Veiga J., Bores-Cerezal A., Palacios-Aguilar J. (2017). Prehospital Emergency Medicine at the Beach: What Is the Effect of Fins and Rescue Tubes in Lifesaving and Cardiopulmonary Resuscitation After Rescue?. Wilderness Environ. Med..

[40] Flouris A.D., Schlader Z.J. (2015). Human behavioral thermoregulation during exercise in the heat. Scand. J. Med. Sci. Sports.

[41] Havenith G., Fogarty A., Bartlett R., Smith C.J., Ventenat V. (2008). Male and female upper body sweat distribution during running measured with technical absorbents. Eur. J. Appl. Physiol..

